# Prenatal Sacrococcygeal Teratoma Diagnosed in a Fetus with Partial Trisomy 13q22

**DOI:** 10.1155/2019/2892869

**Published:** 2019-04-07

**Authors:** Shana S. Dalal, Teresa Berry, Veronica Maria Pimentel

**Affiliations:** ^1^Frank H. Netter MD School of Medicine at Quinnipiac University, North Haven, CT, USA; ^2^Saint Francis Hospital and Medical Center, Hartford, CT, USA; ^3^University of Connecticut School of Medicine, CT, USA

## Abstract

Sacrococcygeal teratoma is a rare neoplasm that arises from a totipotent stem cell in Henson's node. It has rarely been associated with chromosomal abnormalities. We present a unique case of a 25-year-old primigravida at 19 weeks and 5 days of gestation found to have an exophytic complex mass with cystic and solid components in the sacral region. This mass was consistent with a sacrococcygeal teratoma. The patient had originally declined genetic screening. After the ultrasound and genetic counseling, she opted to have cell-free fetal DNA screening that was positive for Trisomy 13. Amniocentesis was performed to confirm the diagnosis. The karyotype demonstrated an abnormality of chromosome 13 and microarray demonstrated a complex structural abnormality of chromosome 13 with large regions of copy number gain. The patient underwent a dilation and evacuation at 23 weeks and 2 days. No fetal autopsy was done. This is a case of a prenatally diagnosed sacrococcygeal teratoma associated with Trisomy 13. It illustrates the diagnostic importance of amniocentesis in setting of fetal anatomical abnormalities on ultrasound. For patients who are reluctant to undergo amniocentesis, cell-free DNA results may provide the additional evidence of the need for diagnostic tests.

## 1. Introduction

Sacrococcygeal teratoma (SCT) is a rare neoplasm that arises from a totipotent stem cell in Henson's node. It occurs in about one out of 40,000 live births and has rarely been associated with chromosomal abnormalities.

## 2. Case Presentation

**Figure 1:**
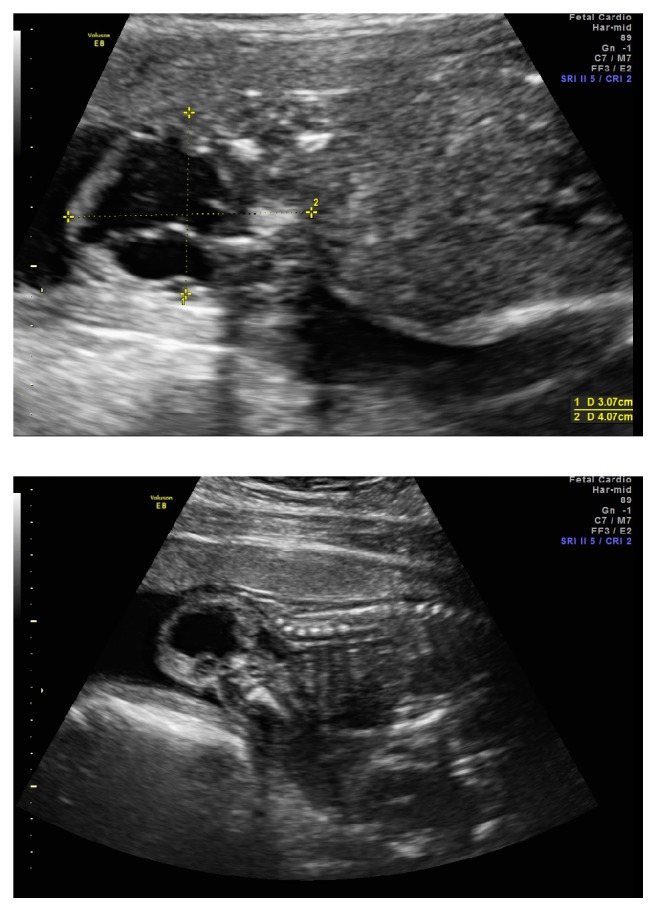
(a) Dimensions of the exophytic mass, measuring 3.07 cm in length and 4.07 cm in width. (b) Exophytic complex mass located in the fetal sacral region with otherwise normal-appearing spine.

We present a case of a 25-year-old primigravida who presented at 19 weeks and 5 days of gestation for an anatomy scan. She had declined genetic screening. An exophytic complex mass with cystic and solid components consistent with that of a sacrococcygeal teratoma (SCT) was found in the sacral region (Figures 1(a) and 1(b)). The spine and brain anatomy was otherwise unremarkable. Pyelectasis was also visualized. No other abnormalities were found.

After genetic counseling, the patient opted to have cell-free fetal DNA screening that was positive for Trisomy 13. Amniocentesis was performed to confirm the diagnosis. The karyotype demonstrated an abnormality of chromosome 13, including duplication. Microarray demonstrated a complex structural abnormality of chromosome 13 with large regions of copy number gain (13q11q22.2 size 56.2 Mb duplication and 13q32.3q34 size 15.5 Mb duplication). Alpha fetal protein from the amniotic fluid was normal (0.61 MoM).

## 3. Discussion

We presented here a unique case of prenatally diagnosed partial Trisomy 13 in the setting of sacrococcygeal teratoma. Reports of chromosomal abnormalities in patients with SCT are rare. There have been few cases of chromosomal abnormalities in patients with SCT including partial Trisomy 10q and partial monosomy 17p, partial monosomy 7q/trisomy 2p, and Trisomy 1q, which were diagnosed prenatally [1–3]. However, the authors found no published reports of SCT associated with Trisomy 13 prenatally in the United States. To date, there have been two other cases of SCT associated with Trisomy 13 postnatally outside to the U.S.

In Central Africa a newborn with clinical features of trisomy 13 was found to have a SCT postnatally; no previous ultrasounds were performed during the pregnancy. This was a clinical diagnosis that was not able to be confirmed due to lack of resources, including karyotype and comparative genomic hybridization. This neonate presented with a bilateral cleft lip and palate, hypotelorism, microcephaly and had a large sacrococcygeal mass with a cystic consistency [4]. The features were clinically consistent with Trisomy 13.

In Turkey, a SCT was found in a newborn postnatally and was later on confirmed to have Trisomy 13. The neonate also presented with aplasia cutis, microphthalmia, low-set ears, depressed nasal root, and polydactyly, other commonly associated features with Trisomy 13 [5]. Our fetus, however, did not prenatally present with any of these commonly associated features.  Table 1 compares the three different cases.

There are published cases of Trisomy 13 with teratomas located in parts of the body other than the sacrum. One case included a juxtarectal cystic teratoma found on a 12-day-old girl with a confirmed t (13;22) translocation. Another teratoma was reported on the neck of a 16-week-old fetus with cleft palate and limb malformations; the karyotype demonstrated Trisomy 13 with centric inversion of chromosome 9. Additionally, an intraoral teratoma was seen in a 24 week-old fetus with Trisomy 13. The umbilical cord of a 17-week-old fetus with Trisomy 13 was also found to have a teratoma [6].

There are increased reports of specific types of malignant and benign tumors in the setting of Trisomy 13 compared to other aneuploidy disorders, possibly indicating that genes on chromosome 13 are associated with this tumor profile. Other than germ cell tumors, leukemia, cutaneous tumors, carcinomas, adenomas, and cerebral tumors have been reported in association with Trisomy 13. However, most of these were diagnosed postnatally [6].

Our case brings new evidence regarding the variety of presentations available with Trisomy 13. Sonographic studies have shown that 91% of fetuses affected with Trisomy 13 have at least one or more sonographically detectable abnormalities. Trisomy 13 is associated commonly with midline located malformations that often include the intrauterine growth restriction, the central nervous system, face, neck, renal, cardiac, extremities, and the abdomen. Holoprosencephaly is one of the most common findings associated with Trisomy 13, but was not seen with this fetus [7]. None of the most commonly reported anomalies of Trisomy 13 were found in this fetus.

Microarray of our fetus was consistent with two large areas of duplication in chromosome 13. The finding of partial instead of full Trisomy 13 may be the reason why none of the commonly associated congenital abnormalities were present in this fetus.

This abnormality of chromosome 13 on this fetus may be either a de novo or an inherited unbalanced translocation, either maternal or paternal in origin. Unfortunately, we were unable to obtain genetic results from either the mother or the father of the baby. The patient elected to terminate the pregnancy at another institution at 23 weeks and 2 days and no fetal autopsy was done.

In conclusion, we have presented a unique case of prenatally diagnosed SCT associated with partial Trisomy 13. This case adds to the limited number of published reports of neoplasms associated with Trisomy 13. More importantly, it provides support for an association between SCT and chromosomal abnormality. Thus, this case illustrates the diagnostic importance of amniocentesis in setting of fetal anatomical abnormalities on ultrasound. For patients who are reluctant to undergo amniocentesis, cell-free DNA results may provide the additional evidence of the need for diagnostic tests. Studies are limited regarding the exact specificity and sensitivity of cell-free fetal DNA for Trisomy 13, but a pooled sensitivity is 0.975 and pooled specificity for all three Trisomies, 13, 18, and 21, is 0.999 [8]. Following the finding of fetal anomalies during the second trimester, amniocentesis is recommended. Anmniotic fluid should be sent for karyotype and microarray.

## Figures and Tables

**Table 1:** Cases of Sacrococcygeal Teratoma associated with Trisomy 13.

Study	Time of Diagnosis	Cytogenic Analysis	Other Features	Location	Outcome
Dalal et al. (2019)	Prenatally	Partial Trisomy 13	Pyelectasis^*∗*^	United States	Dilation and Evacuation: 23 weeks and 2 days

Dorum et al. (2016)	Post-natal	Trisomy 13	Renal cysts, PDA, aplasia cutis, microphthalmia, low-set ears, depressed nasal root, and polydactyly	Turkey	Death from Sepsis: postnatal day 18

Lubala et al. (2015)	Post-natal	None- clinically diagnosed	Bilateral cleft palate, hypotelorism, microcephaly	Democratic Republic of the Congo	Death from Sepsis: postnatal day 8

^*∗*^Ultrasound finding as autopsy not done.

## Abbreviations

SCT: Sacrococcygeal teratoma.
